# Towards cardiac and respiratory motion characterization from electrophysiology data for improved real time MR-integration

**DOI:** 10.1186/1532-429X-15-S1-P68

**Published:** 2013-01-30

**Authors:** Sébastien Roujol, Alex Y Tan, Elad Anter, Mark E Josephson, Reza Nezafat

**Affiliations:** 1 Medecine, BIDMC / Harvard Medical School, Boston, MA, USA

## Background

Electro-anatomical voltage mapping (EAM) is an invasive technique used for the identification of ventricular tachycardia (VT) substrate and subsequent guidance of VT ablation [1]. The mapping of VT substrate is very time-consuming procedure, requires highly skilled electrophysiologist, is associated with patient risk and is an invasive procedure. Late gadolinium enhancement (LGE) MRI allows non-invasive evaluation of 3D structure of scar. Therefore, LGE has the potential to identify the VT substrate and can now be integrated in the current clinical platform for guidance of VT ablation as a roadmap. However, fusion of the two imaging modality is very challenging due to respiratory and cardiac motion during the mapping which results in large errors in data fusion. Our aim in this study is to develop a novel algorithm to detect the respiratory and cardiac-induced motion of the mapping catheter during the VT ablation to facilitate integration of LGE MRI to EAM data.

## Methods

**Figure 1:**
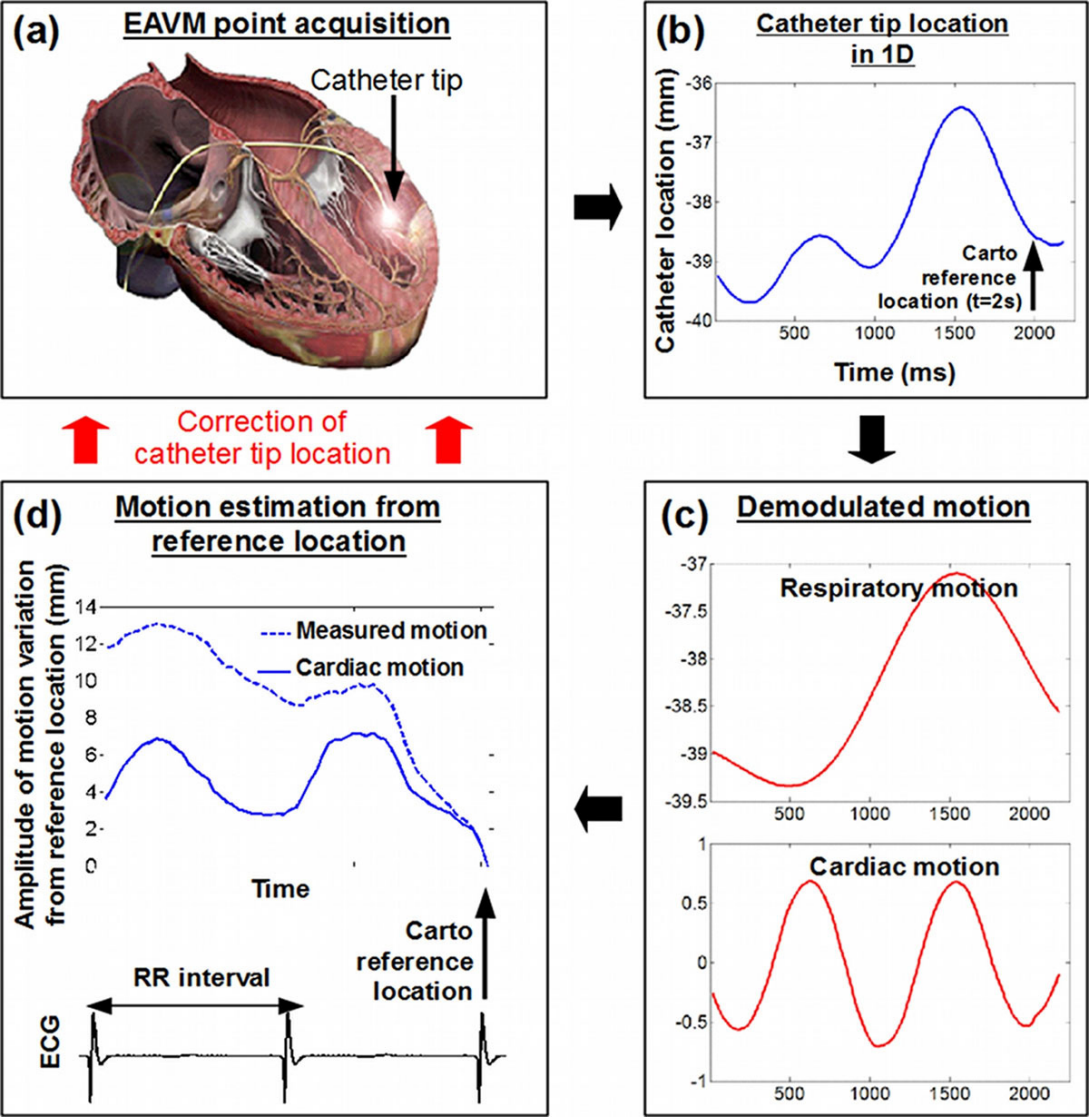
Illustration on real datasets of the cardiac and respiratory motion estimation method. The recorded catheter tip location (b) are frequency filtered to demodulate both cardiac and respiratory motion (c). This then allows to isolate each motion component and estimate the catheter tip location at any respiratory phase or as shown in (d) at any cardiac phase.

The latest EAM navigation platform (Carto 3, Biosense Webster) enables recording of 3D spatial coordinate of the catheter tip for 2.5s for each EAM point. The 3D catheter location is thus influenced by both the contraction of the heart (referred to as cardiac motion) and the breathing activity (referred to as respiratory motion) assuming no operator manipulation of the catheter. In this study, we assume that the respiratory and the cardiac cycles have different frequencies in general in the range of 0.2-0.6Hz and 1-2Hz, respectively. Therefore the respiratory and the cardiac motion are estimated using multiband filters applied to the recorded catheter tip location. Since 2.5s are insufficient to identify a complete respiratory cycle, the estimated respiratory motion of each EAM point was fitted to a respiratory model (a sinus function) in order to estimate the complete respiratory motion pattern. The proposed method is summarized in Figure 1. The method was applied in 17 patients (14 male, 64 years, 108±69 EAM points) undergoing VT ablation for treatment of arrhythmia. 3D catheter locations were extracted for each patient and both respiratory and cardiac motions were estimated.

## Results

**Figure 2:**
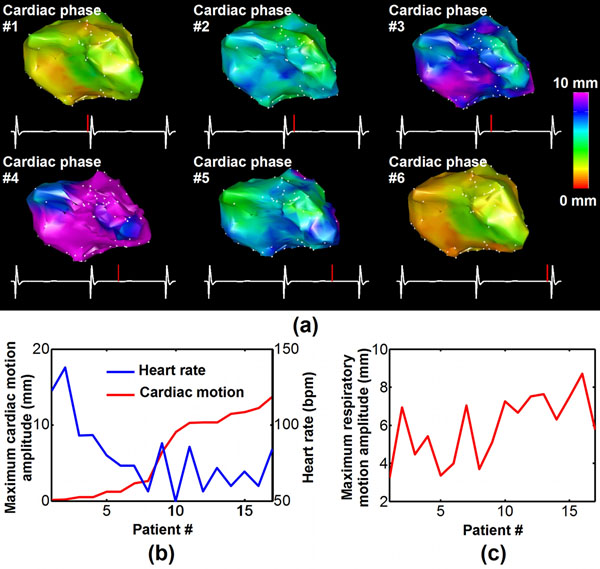
Cardiac and respiratory motion estimated in 17 VT patients. Example of EAM data reconstructed for different cardiac phases based on the estimated cardiac motion (a). The maximum peak to peak amplitude of the estimated cardiac and respiratory motion obtained in 17 VT patients is shown in (b) and (c), respectively.

Example of EAM data reconstructed for different cardiac phases using the estimated cardiac motion is shown in Figure 2a. In average over all patients, the maximum peak to peak amplitude of the estimated cardiac and respiratory motion was found of 6.1±5.1mm and 5.9±1.7 mm, respectively (Figure 2b and 2c). A high inter patient variability was observed for the estimated cardiac motion which was highly correlated with the patient heart rate (r=-0.55, p=0.02).

## Conclusions

The cardiac and respiratory motion can be extracted from 3D catheter location through time. Further studies to integrate the calculated motion to correct for fusion of LGE and MRI are warranted to evaluate the improvement achieved for fusion of the two imaging modalities.

## Funding

NIH:R01EB008743-01A2
